# Results from clinical phase I study on breast tumor ablation with dedicated breast MR-HIFU system

**DOI:** 10.1186/2050-5736-3-S1-O74

**Published:** 2015-06-30

**Authors:** Floor Knuttel, Roel Deckers, Laura Merckel, Gerald Schubert, Max Kohler, Willem Mali, Chrit Moonen, Bartels W Lambertus, Maurice van den Bosch

**Affiliations:** 1 University Medical Center Utrecht, Utrecht, Netherlands; 2 Philips Healthcare, Vantaa, Finland

## Background/introduction

We have recently conducted a clinical phase I study to assess the safety and spatial accuracy and precision of a newly developed dedicated MR-HIFU system for lateral breast tumor ablation [1]. Here, we report on patient inclusion, treatment efficacy and safety.

## Methods

All treatments were performed on a dedicated breast MR-HIFU system (Philips Healthcare, Vantaa, Finland) integrated with a clinical 1.5-T MRI scanner (Achieva, Philips Healthcare, Best, The Netherlands). Ten female patients with i) pathologically proven invasive breast cancer after large-core needle biopsy and ii) tumor size ≥ 1 cm were included. The patients were under procedural sedation during the complete HIFU procedure. Fat-suppressed segmented Echo Planar Imaging was performed for PRFS-based thermometry. Online correction of the respiration-induced field disturbances was performed by a Look-Up Table (LUT)-based method [2]. Partial tumor ablation was performed to allow for histological analysis of viable *versus* ablated tumor tissue. The number of sonication performed per patient (1-5) and the acoustic power (50-90 W) used for each sonication was variable. Surgery was performed at least 48 h after the MR-HIFU procedure. After surgical resection H&E and cytokeratin 8/18 staining was performed on histological sections. In order to assess the safety of the system i) the skin of the treated breast was carefully evaluated by a physician, ii) patients were asked to report pain scores according to the Numerical Rating Scale, with a score of 0 (no pain) to 10 (worst pain imaginable) and iii) the temperature evolution in the temperature imaging slice positioned on the pectoral muscle was analyzed to evaluate possible unintended heating in the far field during tumor ablation.

## Results and conclusions

Seventeen patients were initially enrolled in the study. Seven patients were excluded or withdrew from the study after a pre-treatment MRI scan.

Finally, 10 patients (8 patients with invasive ductal carcinoma and 2 patients with invasive lobular carcinoma) underwent MR-HIFU ablation. The procedural sedation protocol was improved in the course of the clinical study. This lead to less sonication related motion and involuntary patient motion. In addition, the respiration became more regular and shallower, which improved the quality of MR thermometry. The maximum temperature in the focal point increased with increasing power in each patient. Sonications performed with the same acoustic power in different patients lead to different maximum temperatures in the focal point. Finally, clear thermal damage was observed in the tumor tissue in 5 patients.

The absence of thermal damage in the other patients was due to technical problems that prevented the sonication to reach temperatures inside the tumor leading to tissue damage. In none of the patients, skin redness, skin burns or other signs of skin damage were observed. Nausea and vomiting in the hours after the MR-HIFU procedure were reported as minor adverse events in one patient. Only two patients reported pain (maximum score of 5) after the MR-HIFU treatment. No temperature increases related to the tumor ablation were observed in the pectoral muscle during sonication. In conclusion, breast tumor ablation with the dedicated breast MR-HIFU system is safe and technical feasible. A good sedation protocol during HIFU ablation is essential for the success of the treatment.
